# Understanding Rapid Adjustments to Diverse Forcing Agents

**DOI:** 10.1029/2018GL079826

**Published:** 2018-11-08

**Authors:** C. J. Smith, R. J. Kramer, G. Myhre, P. M. Forster, B. J. Soden, T. Andrews, O. Boucher, G. Faluvegi, D. Fläschner, Ø. Hodnebrog, M. Kasoar, V. Kharin, A. Kirkevåg, J.‐F. Lamarque, J. Mülmenstädt, D. Olivié, T. Richardson, B. H. Samset, D. Shindell, P. Stier, T. Takemura, A. Voulgarakis, D. Watson‐Parris

**Affiliations:** ^1^ School of Earth and Environment University of Leeds Leeds UK; ^2^ Rosenstiel School of Marine and Atmospheric Science University of Miami Miami FL USA; ^3^ CICERO Center for International Climate and Environmental Research in Oslo Oslo Norway; ^4^ Met Office Hadley Centre Exeter UK; ^5^ Institut Pierre‐Simon Laplace, CNRS/Sorbonne Université Paris France; ^6^ NASA Goddard Institute for Space Studies New York NY USA; ^7^ Center for Climate Systems Research Columbia University New York NY USA; ^8^ Max‐Planck‐Institut für Meteorologie Hamburg Germany; ^9^ Department of Physics Imperial College London London UK; ^10^ Grantham Institute – Climate Change and the Environment Imperial College London London UK; ^11^ Canadian Centre for Climate Modelling and Analysis Victoria British Columbia Canada; ^12^ Norwegian Meteorological Institute Oslo Norway; ^13^ NCAR/UCAR Boulder CO USA; ^14^ Institute of Meteorology Universität Leipzig Leipzig Germany; ^15^ Nicholas School of the Environment Duke University Durham NC USA; ^16^ Atmospheric, Oceanic and Planetary Physics, Department of Physics University of Oxford Oxford UK; ^17^ Kyushu University Fukuoka Japan

**Keywords:** rapid adjustments, radiative forcing, PDRMIP, kernels

## Abstract

Rapid adjustments are responses to forcing agents that cause a perturbation to the top of atmosphere energy budget but are uncoupled to changes in surface warming. Different mechanisms are responsible for these adjustments for a variety of climate drivers. These remain to be quantified in detail. It is shown that rapid adjustments reduce the effective radiative forcing (ERF) of black carbon by half of the instantaneous forcing, but for CO_2_ forcing, rapid adjustments increase ERF. Competing tropospheric adjustments for CO_2_ forcing are individually significant but sum to zero, such that the ERF equals the stratospherically adjusted radiative forcing, but this is not true for other forcing agents. Additional experiments of increase in the solar constant and increase in CH_4_ are used to show that a key factor of the rapid adjustment for an individual climate driver is changes in temperature in the upper troposphere and lower stratosphere.

## Key Points

- Rapid adjustments affect the Earth's energy balance in different ways for greenhouse gas, aerosol, and solar forcing
- Radiative kernels and partial radiative perturbations are used to diagnose rapid adjustments from atmospheric and cloud changes
- Noncloud adjustments agree well between models, whereas cloud adjustments exhibit more spread

## 1 Introduction

Owing to its expected better correspondence to equilibrium surface temperature change, effective radiative forcing (ERF) has taken precedence over the older definition of stratospherically adjusted radiative forcing (RF) for measuring perturbations to the Earth's radiative energy budget (Boucher et al., 2013; Forster et al., 2016; Myhre et al., 2013; Shine et al., 2003). ERF takes into account the radiative effects of tropospheric and land surface changes to the top of atmosphere (TOA) energy budget, in addition to the stratospheric temperature change, in response to a forcing. Together, these atmospheric and land surface changes are termed rapid adjustments. While there is no formal separation of timescales, rapid adjustments tend to manifest themselves on a period of weeks to months and are distinct from the (generally) slower climate feedbacks, such as the sea ice/albedo, lapse rate, water vapor, and cloud feedbacks, which are driven by the surface temperature response (Sherwood et al., 2015). Therefore, rapid adjustments are usually considered to be part of the forcing (Forster et al., 2013; Gregory et al., 2004).

It has long been known (Manabe & Wetherald, 1975) that an increase in CO_2_ concentrations cools the stratosphere, which reduces longwave (LW) outgoing radiation, increasing the positive forcing compared to the instantaneous radiative forcing (IRF) of CO_2_ (the convention in this paper is to report IRF at the TOA). Such knowledge is incorporated into the definition of RF. More recently, it has been acknowledged that CO_2_ perturbations also change the thermal structure of the troposphere, which leads to changes in water vapor and cloud profiles (Gregory & Webb, 2008). CO_2_ forcing also drives land‐surface changes due to its effects on plant stomatal conductance (Doutriaux‐Boucher et al., 2009; Richardson et al., 2018). These components all induce their own responses in the TOA energy balance, which are not included in the standard RF framework.

Furthermore, for some forcing agents, stratospheric temperatures do not change in the same way as for CO_2_ forcing, and tropospheric changes can be more important (Hansen et al., 2005). Aerosols have significant impacts on tropospheric radiative heating rates and many aspects of clouds (Ramanathan et al., 2001), rendering RF as an unsatisfactory method of comparing forcing impacts across greenhouse gases and aerosols (Boucher et al., 2013; Myhre et al., 2013). To date, rapid adjustment analyses have largely been based on CO_2_ forcing (Andrews & Forster, 2008; Block & Mauritsen, 2013; Chung & Soden, 2015a, 2015b; Gregory & Webb, 2008; Vial et al., 2013; Zelinka et al., 2013) and an investigation across forcing mechanisms has not been made.

## 2 Methods

### 2.1 Experiments and Climate Models

In this study we use atmosphere‐only integrations from 11 global climate models (Canadian Earth System Model version 2 [CanESM2], ECHAM6‐HAM2, Goddard Institute for Space Studies E2‐R [GISS‐E2‐R], Hadley Centre Global Environmental Model version 2 Earth System [HadGEM2‐ES], Hadley Centre Global. Environment Model 3 [HadGEM3], Institut Pierre‐Simon Laplace Coupled Model version 5A [IPSL‐CM5A], Model for Interdisciplinary Research on Climate / Spectral Radiation‐Transport Model for Aerosol Species [MIROC‐SPRINTARS], National Center for Atmospheric Research‐Community Earth System Model version 1‐Community Atmospheric Model version 4 [NCAR‐CESM1‐CAM4], NCAR‐CESM1‐CAM5, Max‐Planck‐Institute Earth System Model [MPI‐ESM], and Norwegian Earth System Model 1‐Medium Resolution [NorESM1]) participating in the Precipitation Driver Response Model Intercomparison Project (PDRMIP; Myhre et al., 2017; Samset et al., 2016) for five idealized climate forcing experiments (Table S1 in the supporting information). The experiments are a doubling of CO_2_ concentrations (2xCO_2_), tripling of methane concentrations (3xCH_4_), 5 times sulfate emissions or concentrations (5xSul), 10 times black carbon emissions or concentrations (10xBC), and a 2% increase in the solar constant (2%Sol). One control (base) integration is also performed for each model. Perturbations are made abruptly, and for each model years 6 to 15 of the control and perturbed integrations are used for analysis. For the 5xSul and 10xBC experiments, some models are driven by emissions rather than concentration increases, and aerosol mass loadings depend on the aerosol transport and dynamical schemes within the model. Therefore, the emission‐driven experiments are not necessarily equal in terms of forcing size perturbation with each other or with the concentration‐driven experiments (Figures S1 and S2 in supporting information).

The atmosphere‐only integrations use climatological sea surface temperatures (SSTs) and sea ice. ERF is defined as the difference in TOA flux imbalance between the perturbed and base integrations of each climate model using the same base SST and sea ice climatology in both runs. By fixing the ocean state, any contribution from climate feedback is minimized leaving just the IRF plus the rapid adjustments, which sums to the ERF (Forster et al., 2016). For aerosol experiments, we define IRF as the sum of the direct aerosol effect (RFari) and cloud‐albedo effect (RFaci), whereas adjustments include the semidirect and cloud‐lifetime effects (Boucher et al., 2013). Land surface temperatures are allowed to respond, and feedback from land surface temperature changes, although small, do get aliased into calculations of adjustments (Chung & Soden, 2015b). There is more than one way to define ERF (Gregory et al., 2004; Hansen et al., 2005; Myhre et al., 2013), but we use the fixed‐SST method as fewer model years are needed to minimize the uncertainty in ERF (Forster et al., 2016).

### 2.2 Radiative Kernel Method

Assuming that rapid adjustments are sufficiently linear and separable, radiative kernels can be used to diagnose adjustments. Radiative kernels describe how the TOA radiative flux changes for a small perturbation in an atmospheric state variable. The kernel technique has generally been used for studies quantifying climate feedback in coupled model integrations (Block & Mauritsen, 2013; Shell et al., 2008; Soden et al., 2008) but is also useful for diagnosing rapid adjustments by applying them to atmosphere‐only integrations (Chung & Soden, 2015a; Vial et al., 2013; Zhang & Huang, 2014).

We can write the ERF resulting from a small perturbation in climate as (Chung & Soden, 2015a)

(1) $$\mathrm{ERF}=\mathrm{IRF}+A_{T}+A_{T_{s}}+A_{q}+A_{\alpha }+A_{c}+\epsilon ,$$

where *A*_*x*_ is the rapid adjustment *x* due to atmospheric temperature (*T*), surface temperature (*T*_*s*_), water vapor (*q*), surface albedo (*α*), and clouds (*c*), and *ϵ* is a residual that accounts for nonlinearities. The rapid adjustment due to atmospheric temperature is further broken down into stratospheric and tropospheric contributions, using a tropopause that varies linearly from 100 hPa at the equator to 300 hPa at the poles (Soden et al., 2008). In this study we use radiative kernels from the Bureau of Meteorology Research Centre (BMRC; Soden et al., 2008), Community Climate System Model version 4 (CCSM4; Shell et al., 2008), CESM (Pendergrass et al., 2018), Geophysical Fluid Dynamics Laboratory (GFDL; Soden et al., 2008), HadGEM2 (Smith, 2018), and European Centre for Medium‐Range Weather Forecasts (ECMWF)‐ERA‐Interim/Oslo (Dee et al., 2011; Myhre et al., 2018; Myhre & Stordal, 1997) models.

As cloud adjustments do not typically behave linearly following a perturbation in cloud properties, the adjustments due to clouds are calculated differently. One method is using the difference of all‐sky and clear‐sky kernel decompositions:

(2) $$A_{c}=\left(\mathrm{ERF}-\mathrm{ER}F^{\mathrm{clr}}\right)-\left(\mathrm{IRF}-\mathrm{IR}F^{\mathrm{clr}}\right)-\sum_{x\in \left\{T,q,\alpha ,T_{s}\right\}}\left(A_{x}-{A_{x}}^{\mathrm{clr}}\right)$$

where ERF^clr^ and IRF^clr^ refer to cloud‐free ERF and IRF and $A_{x}^{\mathrm{clr}}$ are rapid adjustments calculated with clear‐sky kernels. We refer to this as the kernel difference method. IRF and IRF^clr^ are not known precisely from many models, but in some cases an estimate can be made by substituting each model's base and perturbed climatology into an off‐line radiation code and taking the difference in TOA fluxes.

### 2.3 Monthly Mean Partial Radiative Perturbation Method

The ECMWF‐ERA‐Interim/Oslo kernel did not implement a water vapor kernel, and water vapor and cloud adjustments using this radiation code were computed using a variant of the Partial Radiative Perturbation (PRP) method (Colman et al., 2001; Wetherald & Manabe, 1988), which we denote Monthly Mean Partial Radiative Perturbation (MMPRP) as it uses monthly mean climatologies from each perturbed run. Water vapor and cloud profiles from each experiment are substituted into the control climatology, run through an off‐line radiative transfer model and the difference in TOA fluxes computed compared to the control climatology. As the MMPRP does not require knowledge of the IRF, cloud adjustment calculations are available from more models in the 10xBC and 5xSul experiments than with the kernel difference method. The SW cloud adjustments for 5xSul were further decomposed using the Approximate Partial Radiative Perturbation (APRP) method (Taylor et al., 2007).

Further methodological details describing the radiative kernel, MMPRP and APRP methods are provided in the supporting information (Alduchov & Eskridge, 1996; Collins et al., 2006; Edwards & Slingo, 1996; Huang & Bani Shahabadi, 2014; Manners et al., 2015; Martin et al., 2011; Sanderson & Shell, 2012; Zelinka et al., 2014).

## 3 Results

### 3.1 Rapid Adjustments by Forcing Agent

**Figure 1:**
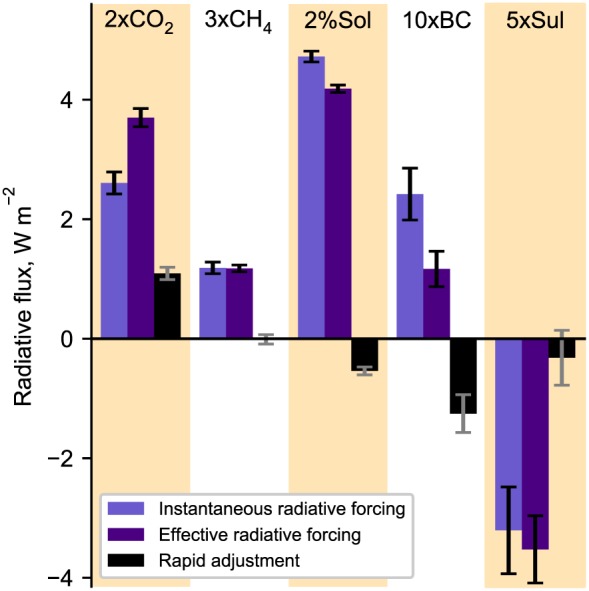
Instantaneous radiative forcing, effective radiative forcing, and rapid adjustments for five different climate change drivers. Each bar represents a multimodel mean and each model is given equal weight with up to six different methods of calculating each adjustment for each model (Figure S3 in the supporting information). The error bars are the 95% confidence range weighting each method and model sample equally.

Figure 1 shows the IRF, ERF, and rapid adjustments for each of the five climate forcers. The rapid adjustments to 2xCO_2_ forcing are positive overall and constitute 30% of the ERF, whereas for 10xBC forcing they are negative and are 107% of the absolute ERF, therefore offsetting more than half of the positive IRF. Rapid adjustments to 2%Sol and 5xSul are slightly negative, whereas they are close to zero for 3xCH_4_.

Adjustments are calculated individually for each model and kernel (Figure S3 in the supporting information), and the responses are averaged first over kernels and then over models so that each model has equal weight in Figure 1. In previous work it has been shown that differences in kernel base climatology and radiative transfer are typically minimal (Soden et al., 2008), and differences are only significant where climate perturbations are larger than in this study (Jonko et al., 2012). In general, we find this to be true as interkernel agreement is good except for stratospheric temperature adjustment to 2xCO_2_ forcing as discussed below. Interkernel agreement is also good for cloud adjustments, although cloud adjustments calculated using MMPRPs differ somewhat from the kernel‐derived methods. For 5xSul we only use cloud adjustments calculated from the MMPRP for Figure 1 except from the NCAR‐CESM1‐CAM4 model, which does not include cloud microphysical changes in its PDRMIP configuration. This is to isolate the component of the cloud lifetime effect (Albrecht, 1989) from the cloud microphysical effect (Twomey, 1977).

**Figure 2:**
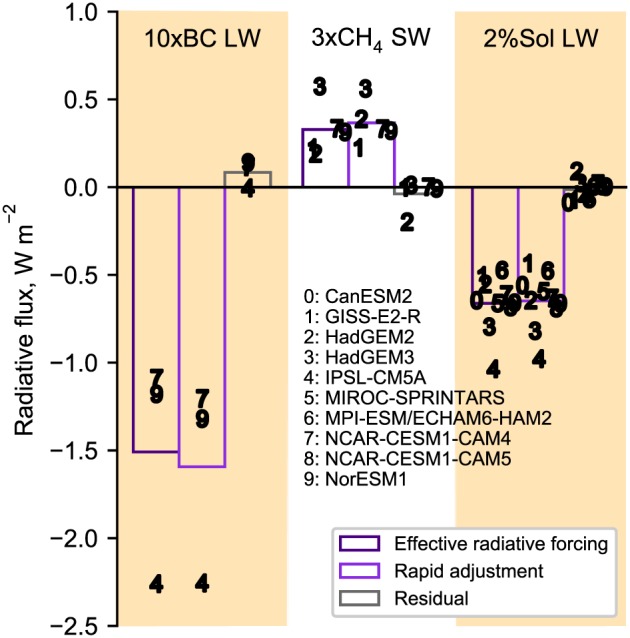
Effective radiative forcing, rapid adjustments, and residuals in models where instantaneous radiative forcing is known to be exactly 0. Each point is a multikernel mean for each model and outlined bars show the multimodel means from this subset of models.

The residual term ***ϵ*** in equation (1) should be no more than 10% of the ERF for the kernel method to be valid (Shell et al., 2008). For most models and experiments the true IRF is not available as this requires a double radiation call. However, in some situations the shortwave (SW) or LW IRF is known to be 0. This occurs when either SW or LW absorption of a particular species is not present in a model's radiation code. In these cases, any difference between the ERF and the sum of all rapid adjustments is a residual by equation (1). Figure 2 shows the ERF, rapid adjustment, and residual for 10xBC LW, 3xCH_4_ SW, and 2%Sol LW for those models, which do not include LW absorption of BC or SW absorption of methane in their radiation schemes (no models include LW solar absorption). In each of the three cases it can be seen that the residual term is small, being 6%, 12%, and 2% of the ERF for 10xBC LW, 3xCH_4_ SW, and 2%Sol LW in magnitude, respectively. The larger multimodel residual in the 3xCH_4_ SW case is biased by a large relative residual in the HadGEM2 model, whereas residuals in the other four models analyzed are close to 0.

**Figure 3:**
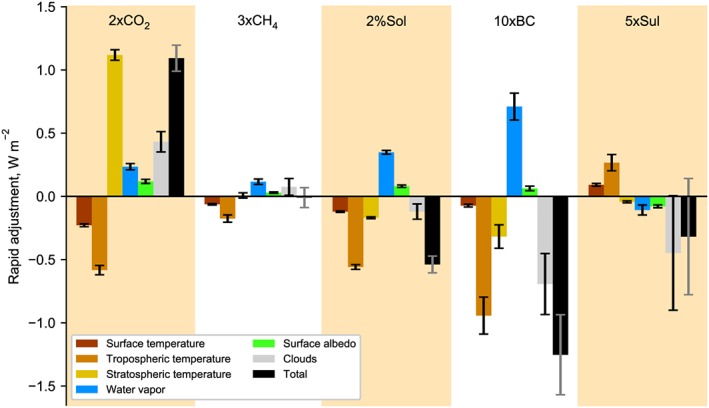
Rapid adjustments decomposed by mechanism. The bars represent multimodel means. The error bars are the 95% confidence range weighting each method and model sample equally (see Figure S3 for individual model and kernel responses).

Figure 3 shows the contribution to the total rapid adjustment for each atmospheric mechanism. Adjustments normalized by IRF and the mean change in individual model atmospheric profiles are shown in Figures S4 and S5 in the supporting information.

For 2xCO_2_, the adjustment due to stratospheric temperature change is strong and positive due to stratospheric cooling. The stratospheric cooling is the largest temperature response throughout the atmospheric column and is consistently present in all models. It is a remarkable coincidence that the stratospheric temperature adjustment for 2xCO_2_ is the same size as the total adjustment, despite the nonnegligible contributions from other adjustments, which sum to 0. This can explain why stratospherically adjusted RF has for a long time been a useful metric for evaluating the energetic effects of CO_2_ forcing. The larger error bar for the total adjustment reflects the assessment of Myhre et al. (2013) that ERF is expected to be similar to RF for CO_2_ forcing with a larger uncertainty due to the inclusion of the tropospheric and surface adjustments.

There is some disagreement between kernels for the stratospheric temperature adjustment for 2xCO_2_, for which the ECMWF‐ERA‐Interim/Oslo kernel projects a stronger adjustment in most models. The reason for this is that the ECMWF‐ERA‐Interim/Oslo kernel has a higher resolution in the stratosphere than the other kernels considered in this study. The ECMWF‐ERA‐Interim/Oslo kernel has 60 model levels with the highest at 0.1 hPa, whereas the other five kernels use the 17 standard pressure levels in CMIP5 (Taylor et al., 2012) with 10 hPa being the highest. The difference is largest in models for which the output is also highly resolved in the stratosphere, such as CanESM2. This additional contribution to the stratospheric cooling accounts for about an additional +0.4 W/m^2^ allocated to stratospheric adjustment in CanESM2 using the ECMWF‐ERA‐Interim/Oslo kernel compared to other kernels.

Black carbon shows a strong negative total adjustment (Stjern et al., 2017), for which the largest (absolute) negative contributions are tropospheric temperature and clouds. The largest positive adjustment in absolute terms is due to the change in water vapor, which opposes tropospheric temperature changes. The vertical profiles associated with BC forcing show a warming and wetting throughout the atmosphere coupled with a large reduction in cloud fraction, particularly at higher levels. Unlike for 2xCO_2_, the stratospheric temperature adjustment is negative rather than positive in 10xBC, due to a warming stratosphere (Hansen et al., 1997). The total adjustment is substantially different from the stratospheric temperature adjustment, so RF is not an appropriate measure of ERF for BC forcing. The strong negative adjustment to black carbon forcing indicates that the equilibrium global mean near‐surface temperature response to a black carbon forcing is smaller than indicated by its direct effect. Unlike CO_2_, BC (and sulfate) mass mixing ratios are spatially variable, and rapid adjustments depend on the location of the aerosol perturbation (Samset & Myhre, 2015). An increase in solar forcing is not distributed equally either, as more solar radiation is received in the tropics, and this zonal asymmetry can drive different circulation responses compared to CO_2_ (Guo et al., 2018).

In the 2%Sol experiment, the tropospheric temperature adjustment (partially offset by water vapor) also dominates over the stratospheric temperature adjustment. Net cloud adjustments are negative rather than positive for 2%Sol as seen by the change in vertical cloud fraction profile but are smaller in magnitude relative to the IRF than for 10xBC. Again, the rapid adjustment due to stratospheric temperature change does not equal the total adjustment.

The stratospheric temperature adjustment in 3xCH_4_ shows a much smaller level of change than 2xCO_2_, as stratospheric temperature changes are much less pronounced. The small stratospheric temperature adjustment could be due to competition between radiative absorption and emission processes in the upper troposphere/lower stratosphere (UTLS; Zhong et al., 1993), unlike for CO_2_ in which emission from the upper troposphere dominates due to its relatively higher abundance in the atmosphere (Forster et al., 1997; Stocker et al., 2001). Therefore, for 3xCH_4_, more of the LW emission from the warming troposphere passes through the stratosphere and reaches the TOA. However, overlap with water vapor bands for CH_4_ is also important. The subset of models with an explicit treatment of SW absorption by methane (CanESM2, MIROC‐SPRINTARS, MPI‐ESM, and NCAR‐CESM1‐CAM5) exhibits a UTLS warming and a negative stratospheric temperature adjustment to methane forcing; the opposite is true for those that do not (Figures S5b and S6 in the supporting information). Inclusion or omission of SW methane absorption also determines whether cloud adjustments are negative or positive. In the multimodel mean, the stratospheric temperature adjustment is similar to the total adjustment, but for the subset of models that either exclude or include SW absorption of methane, they do not agree. This may indicate that ERF also differs from RF for methane.

In 5xSul, noncloud adjustments are generally small in comparison to the IRF, and cloud adjustments, which are larger in magnitude and spread, are described in the following subsection.

In all experiments there is an anticorrelation between tropospheric temperature and water vapor adjustments, which is a result of the Clausius‐Clapeyron relationship. This is shown by observing that the water vapor adjustment is dominated by the adjustment calculated assuming a constant relative humidity for the tropospheric temperature change in each model and experiment (Figure S7 in the supporting information). By decomposing tropospheric temperature responses into Planck and lapse‐rate components, we also show that the model spread in lapse‐rate plus constant relative humidity is smaller than in each of the individual components, reinforcing this anticorrelation and in line with feedback studies (Soden et al., 2008).

### 3.2 Cloud Adjustments

**Figure 4:**
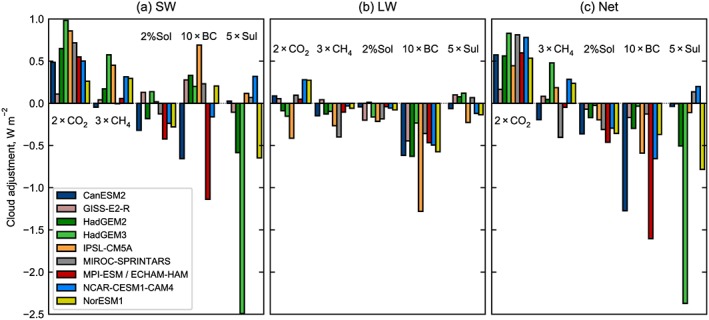
Cloud adjustments in each model using the Monthly Mean Partial Radiative Perturbation method. National Center for Atmospheric Research‐Community Earth System Model version 1‐Community Atmospheric Model version 5 (NCAR‐CESM1‐CAM5) is excluded as not all required diagnostics are present.

The cloud adjustments in each model and forcing agent are further split into SW and LW components in Figure 4 using the MMPRP method. Cloud adjustments to CO_2_ forcing are +0.45 W/m^2^ on average and all models and methods agree that the net effect is positive, dominated by the SW effect. This is due to a reduction in low‐level and midlevel cloud fraction (also noted in Zelinka et al., 2013), which reduces outgoing SW radiation. There is no agreement between models on the sign of the LW cloud adjustment.

In 10xBC, a downward shift of clouds occurs. Many competing processes that affect cloud adjustments occur in response to a BC forcing, such as cloud burn off (reduction in clouds), increased convection (increasing clouds), and changes in atmospheric stability, which could increase or decrease clouds depending on the altitude of the BC perturbation (Koch & Del Genio, 2010; Samset & Myhre, 2015). Every model shows a negative LW cloud adjustment to 10xBC, whereas there is no agreement on the sign of SW cloud adjustments. However, those models with a substantial negative cloud adjustment to 10xBC (ECHAM6‐HAM2 and CanESM2) show a large increase in cloud liquid water content in the lower or middle troposphere that is not present in other models, suggesting a possible mechanism.

For 5xSul, there is a large spread in the strength of the cloud adjustment, dominated by the SW effect, which is particularly strong in the HadGEM3 model. Further analysis is undertaken with the APRP method for SW cloud adjustments in the seven models for which diagnostics permit (Figure S8 in supporting information). This shows that the ERF for 5xSul is substantially more negative in HadGEM3 than for other models, driven by a large contribution from the RFaci (RF due to aerosol‐cloud interactions or cloud microphysical effect). The direct aerosol forcing (RF due to aerosol‐radiation interactions, RFari) in HadGEM3 is in line with other models. The APRP highlights the varying strength in RFaci, which ranges from 33% (in CanESM2) to 63% (in HadGEM3) of the ERF in models, which include cloud microphysical changes.

One limitation of the APRP method is that it does not appear to work well with models that do not include interactive cloud microphysical changes in their configurations, such as the apparent positive RFaci in NCAR‐CESM1‐CAM4 and GISS‐E2‐R. Furthermore, the computed SW cloud adjustments with the MMPRP and APRP methods do not agree in magnitude, and the kernel difference method, which is only available in three models due to the availability of double calls, only agrees with the MMPRP where cloud microphysical changes are not included (i.e., in NCAR‐CESM1‐CAM4; Figure S3). The differences obtained with different methods highlight the difficulties in estimating cloud adjustments. A direct estimate of cloud adjustments can be obtained using a cloud kernel (Zelinka et al., 2012) and model output of International Satellite Cloud Climatology Project (ISCCP) simulator diagnostics (Bodas‐Salcedo et al., 2011; Klein & Jakob, 1999; Webb et al., 2001). These diagnostics were not part of the PDRMIP data request and are generally not output by models outside of the CFMIP protocol (Webb et al., 2017). Inclusion of these diagnostics as standard in more models would aid understanding of cloud adjustment mechanisms.

## 4 Conclusions

Understanding how models respond to different forcing agents is critical to reducing uncertainty in climate‐model projections of the Earth's energy budget and ultimately climate sensitivity (Forster et al., 2016). This work has found that ERF is distinctly different from RF across forcing mechanisms, indicating that for all forcings analyzed rapid adjustments have an important role to play in the quantification of the perturbation of the Earth's energy budget from human activities and the overall heating of the oceans. For CO_2_ competing tropospheric and land surface rapid adjustment effects cancel, meaning the overall estimates of ERF are similar to the stratospheric temperature adjusted RF. For the other mechanisms tropospheric rapid adjustments significantly alter forcing estimates. Tropospheric temperature (partially offset by water vapor) and cloud changes are the principal sources of adjustment, with stratospheric temperature adjustment playing a lesser role. Clouds have an appreciable effect on the overall forcings, particularly for CO_2_, BC, and sulfate, and it is shown that intermodel diversity is high and diagnostic methods do not necessary agree. For CH_4_ the sign of the cloud adjustment, and total rapid adjustment, depends on whether SW absorption of methane is included in the radiation scheme. This highlights the importance of the UTLS region in the radiative balance and has important consequences for model development.

The vertical resolution and truncation height of both the radiative kernel and model output may affect the rapid adjustment due to stratospheric temperature calculated for CO_2_ forcing. It may be the case that adjustments or climate feedbacks are underestimated using radiative kernels or model output, which is not well resolved in the stratosphere. As radiative kernels have been used extensively for multiple‐CO_2_ studies or anthropogenic forcing pathways where CO_2_ is the dominant forcing agent, further work is needed to determine whether this is a significant effect.

In general, noncloud adjustments agree well between methods and models, and radiative kernels provide a robust framework for decomposing ERF into IRF and rapid adjustments. However, cloud adjustments are not consistent between models and methods, particularly for sulfate forcing, and require more understanding. Further modeling work should focus on improving our understanding of cloud adjustments, and output of ISCCP simulator diagnostics as standard in climate models would be beneficial to enable a consistent method of directly estimating these effects. A further progression is to constrain rapid adjustment calculations with observations. A historical reanalysis data set can be used to determine the evolution of rapid adjustments over time. In the present day, satellite and ground‐based stations can be used to observationally constrain the spatial distribution of aerosol concentrations, which would be particularly important for black carbon where the adjustment strength (and possibly sign) is sensitive to the vertical profile of the aerosol.

## Supporting information



Supporting Information S1
