# Supplementary material for: Understanding Rapid Adjustments to Diverse Forcing Agents
Source: Geophys Res Lett. 2018 Nov 8;45(21):12023–31. doi: 10.1029/2018GL079826 (PMC6334512; doi:10.1029/2018GL079826)
Supplement: Supplementary file 1 — Supporting Information S1 [file GRL-45-12023-s001.docx]

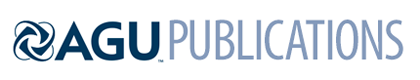


*Geophysical Research Letters*

Supporting Information for

**Understanding Rapid Adjustments to Diverse Forcing Agents**

C.J. Smith^1^, R.J. Kramer^2^, G. Myhre^3^, P.M. Forster^1^, B. Soden^2^, T. Andrews^4^, O. Boucher^5^, G. Faluvegi^6,7^, D. Fläschner^8^, Ø. Hodnebrog^3^, M. Kasoar^9,10^, V. Kharin^11^, A. Kirkevåg^12^, J.-F. Lamarque^13^, J. Mülmenstädt^14^, D. Olivié^12^, T. Richardson^1^, B.H. Samset^3^, D. Shindell^15^, P. Stier^16^, T. Takemura^17^, A. Voulgarakis^9^, D. Watson-Parris^16^

1. School of Earth and Environment, University of Leeds, Leeds, LS2 9JT, UK
2. Rosenstiel School of Marine and Atmospheric Science, University of Miami, 4600 Rickenbacker Causeway, Miami, FL 33149, USA
3. CICERO Center for International Climate and Environmental Research in Oslo, 0318, Oslo, Norway
4. Met Office Hadley Centre, Exeter, Devon, EX1 3PB, UK
5. Institut Pierre-Simon Laplace, CNRS/Sorbonne Université, 75252, Paris, Cedex 05, France
6. NASA Goddard Institute for Space Studies, New York, NY, 10025, USA
7. Center for Climate Systems Research, Columbia University, New York, NY, 10027, USA
8. Max-Planck-Institut für Meteorologie, 20146, Hamburg, Germany
9. Department of Physics, Imperial College London, London, SW7 2AZ, London, UK
10. Grantham Institute – Climate Change and the Environment, Imperial College London, SW7 2AZ, UK
 11. Canadian Centre for Climate Modelling and Analysis, V8P 5C2, Victoria, BC, Canada
12. Norwegian Meteorological Institute, 0313, Oslo, Norway
13. NCAR/UCAR, 80305, Boulder, CO, USA
14. Institute of Meteorology, Universität Leipzig, 04107 Leipzig, Germany
15. Nicholas School of the Environment, Duke University, 27708, Durham, NC, USA
 16. Atmospheric, Oceanic & Planetary Physics, Department of Physics, University of Oxford, Parks Road, Oxford, UK
17. Kyushu University, 816-8580, Kasuga, Fukuoka, Japan

**Contents of this file**

Text S1 to S5

Figures S1 to S10

Table S1

Text S1. Radiative kernel method

The radiative kernel $K_{x}$ approximates the change in TOA radiation $\Delta R$ (SW or LW) due to a unit change in state variable $\Delta x$, such that $K_{x}\approx\partial R/\partial x$ and $A_{x}=K_{x} \Delta x$. $x$ represents atmospheric temperature ($T$), surface temperature ($T_{s}$), specific humidity ($q$) or surface albedo ($\alpha$). Radiative kernels themselves are produced by making small perturbations in a climate model’s base climatology and running the resulting atmosphere through an offline radiative transfer code comparing the TOA fluxes between the perturbed state and the climatology; Text S2 describes this in more detail in construction of the HadGEM2 kernel. Two sets of kernels are produced: clear-sky kernels ${K_{x}}^{\mathrm{clr}}$ where the resulting fluxes are calculated with clouds absent, and all-sky kernels $K_{x}$ (for $x\in T, q, \alpha, T_{s}$). $K_{x}$ and $\Delta x$ are four-dimensional (month, pressure level, latitude, longitude) fields for atmospheric temperature and specific humidity, and three-dimensional (month, latitude, longitude) for surface temperature and surface albedo.

Substituting in the kernel terms for each adjustment, eq. (1) in the main paper can be written as

|  | $ERF=IRF+\frac{\partial R}{\partial T}\Delta T+\frac{\partial R}{\partial\left( \log q \right)}\Delta\log q+\frac{\partial R}{\partial T_{s}}\Delta T_{s}+\frac{\partial R}{\partial\alpha}\Delta\alpha+A_{c}+\epsilon.$ | (S1) |
| --- | --- | --- |

For $T$ and $T_{s}$, the perturbation unit for $\partial R/\partial T$ is 1 K, so the kernel is multiplied by the Kelvin change between the perturbed and base climates. The near-surface air temperature (CMIP5 variable name tas) is used for surface temperature changes, as the skin temperature (CMIP5 name ts) was not output as a diagnostic in all models. Block and Mauritsen (2013) report a difference of around 5% in climate feedback parameter between the two values; the surface temperature adjustments in this study are small, due to our use of fixed-SST integrations, and this difference is not likely to be substantial.

For $\alpha$ the perturbation units are percent (additive) and again the adjustment is linear with the perturbation. As LW and SW absorption scales approximately logarithmically with specific humidity (Huang & Bani Shahabadi, 2014), the calculation is different for water vapour changes (Sanderson & Shell, 2012)

|  | $\Delta(\log q)=\frac{\log q_{\mathrm{PTB}}-\log q_{\mathrm{CTL}}}{\log e_{s}\left( p,T_{a}+1 \right)/\log e_{s}(p,T_{a}) - 1},$ | (S2) |
| --- | --- | --- |

where $e_{s}(p,T_{a})$ is the saturation specific humidity in the base run for the model pressure *p* and temperature $T_{a}$, and the subscripts PTB and CTL represent specific humidity from the perturbation and control runs. The specific humidity kernel $K_{q}$ is defined as the change in TOA flux that maintains a constant relative humidity in the base climate for a 1 K temperature increase (Shell et al., 2008; Soden et al., 2008). Some models did not archive specific humidity, so in these models and in the equation above we use the relationship of Alduchov and Eskridge (1996) to calculate saturation specific humidity $w_{s}$:

|  | $w_{s}=\frac{0.622e_{s}}{p-e_{s}};$ | (S3) |
| --- | --- | --- |

|  | $e_{s}=610.94\exp\left( \frac{17.625\left( T_{a}-273.15 \right)}{T_{a}-30.11} \right).$ | (S4) |
| --- | --- | --- |

This relationship is well-behaved below about 7 hPa. Above this level, the denominator in eq. (S3) is no longer typically an order of magnitude greater than the numerator, and we set all humidity changes above 7 hPa to zero to prevent numerical artefacts. As the upper stratosphere is dry and kernel sensitivity to specific humidity changes is low there, this causes minimal impact to our adjustment calculations.

For 4-dimensional variables, the units of the radiative kernels are W m^-2^ K^-1^ (100 hPa)^-1^. Each vertical level from the model output is first converted to pressure coordinates (if not already expressed in pressure coordinates), multiplied by the kernel, and then multiplied by the thickness of the model level in pressure coordinates divided by 100 hPa. The total adjustment is the sum over each level in the atmosphere. For $A_{x}$ we report horizontal and temporal means, after summation over the vertical dimension if applicable.

Text S2. HadGEM2 radiative kernels

This paper introduces kernels derived from the HadGEM2-ES climate model. The kernels are generated in a similar way to that of others (Block & Mauritsen, 2013; Pendergrass et al., 2018; Shell et al., 2008; Soden et al., 2008).

One year from a fixed-SST pre-industrial run of the HadGEM2-ES climate model (1.875° longitude × 1.25° latitude, on 38 model levels up to 39 km) was performed, with output archived every three hours. The model-generated air temperature on model layers and layer boundaries, surface temperature, specific humidity on layers, cloud water content, cloud ice content and cloud fraction on each model level, and all-sky and clear-sky surface downwelling and upwelling radiation fluxes were archived. Greenhouse gases, including ozone, are set to 1860 levels, and aerosols are not included.

Firstly, the 3-hourly climate base state is run offline through the SOCRATES radiative transfer code, and TOA LW and SW fluxes saved. For consistency with the climate model, the HadGEM2-ES version of SOCRATES (Edwards & Slingo, 1996; Martin et al., 2011) was used. Cloud droplets were taken to have an effective radius of 10 micron for liquid and 30 micron for ice, and surface albedo is calculated as the ratio of surface upwelling to surface downwelling clear-sky shortwave radiation. To build the kernel, the surface temperature, surface albedo, air temperature and specific humidity are perturbed in turn, and top-of-atmosphere (TOA) LW and SW radiation flux differences for each perturbation compared with the control climate state. Surface temperatures are increased uniformly by 1 K. Surface albedo is increased by 1% (absolute). Air temperature is increased by 1 K on each of the 38 model levels in turn, and by 0.5 K on the bounding half-levels. The water vapor kernel is created by perturbing each model layer in turn by an amount in specific humidity that would maintain the same relative humidity for a temperature increase of 1 K, calculated using eqs. (S3) and (S4).

For each three hour time step, a total of 178 offline radiative transfer calculations are performed. This comprises of 38 for air temperature, 38 for water vapor, and one each for surface temperature, surface albedo, and the control state. All calculations are performed for clear-sky and all-sky to generate clear-sky and all-sky kernels. The 3-hourly responses are averaged each month, giving four-dimensional kernels with dimensions time=12, levels=38, latitude=145, longitude=192 for atmospheric temperature and specific humidity (Figure S9), and three-dimensional kernels for surface albedo and surface temperature (Figure S10).

The 38-level model output for temperature and specific humidity is then interpolated to the standard 17 CMIP pressure levels (Taylor et al., 2012). The normalization coefficient to convert from model-derived TOA flux changes in W m^-2^ to W m^-2^ (100 hPa)^-1^ on each layer $i$ is

|  | $\frac{10000}{p_{i+\frac{1}{2}}-p_{i-\frac{1}{2}}};$ | (S5) |
| --- | --- | --- |

where the denominator represents the monthly mean layer thickness on pressure coordinates (i.e. between half levels). After normalization, the interpolation is performed.

In addition to the TOA kernels used in this study, a set of 17- and 38-level surface flux kernels were generated. They are derived in the same way as the TOA kernels, where the flux difference of interest is the net surface flux.

Both the TOA and surface kernels on the 17 CMIP pressure levels and 38 HadGEM2-ES model levels are available from <https://doi.org/10.5518/406>.

Text S3. Instantaneous radiative forcing

Typically the instantaneous radiative forcing (IRF) is not saved from climate model integrations, and has to be estimated in feedback studies (Soden et al., 2008). A number of models in this study have IRFs available diagnosed from a “double call” (Chung & Soden, 2015): a second call to the model radiation scheme with the forcing agent in question set to zero or the pre-industrial value (Table S1). Availability of model-specific IRFs for all-sky and clear-sky conditions allows a complete decomposition of adjustments including calculation of the residual term.

For solar forcing, the IRF was calculated from geometry in all models by

|  | $IRF_{2\%Sol} = 0.02 \times\frac{1}{4} \times S\left( 1-P \right)\boldsymbol{,}$ | (S6) |
| --- | --- | --- |

where $S$ is the solar constant used in each model and $P$ is the planetary albedo in the control integration. Separate calculations for all-sky and clear-sky IRF (using all-sky and clear-sky $P$) are performed.

For 2xCO_2_ and 3xCH_4_, model-specific IRFs were only available for HadGEM2-ES and NCAR-CESM1-CAM4. In other models, they were estimated by running the control and perturbed values of CO_2_ and CH_4_ offline with each model’s control climatology in the SOCRATES radiative transfer code (Edwards & Slingo, 1996; Manners et al., 2015). Although this does not capture the diversity in each model’s radiative transfer parameterization, for which there can be significant differences (Collins et al., 2006), it does account for the contribution to the spread from each model’s base climatology. Owing to the spatial diversity of aerosols and the substantially differing treatment of scattering and absorption of aerosols in different models, no attempt to estimate IRF was made in those models that did not provide double calls in 10xBC and 5xSul. Six models were able to provide double calls for 10xBC, whereas three models saved IRF diagnostics for 5xSul.

In Figure 1 in the main manuscript, IRF is estimated assuming that the residual from the kernel decomposition is zero, including in those models and experiments where IRFs are available from the model or have been estimated. This can be justified by the observation that residuals are small in the cases where IRF is known and is zero (Figure 2). The IRFs calculated as described in this section were used to estimate cloud adjustments using the kernel-difference method, as described in section 2.2 and eq. (3).

Text S4. Monthly mean partial radiative perturbation (MMPRP)

Alongside the kernel method we use an adaptation of the partial radiative perturbation (PRP) described below to estimate water vapor and cloud adjustments in the Oslo code (Colman et al., 2001; Wetherald & Manabe, 1988). As most models produced only monthly mean diagnostics, monthly mean inputs were used in the PRP, and so we define this method as the monthly mean PRP (MMPRP) to distinguish it from a true PRP which uses instantaneous, sub-daily output.

In the MMPRP, the variable of interest is substituted from the perturbation run in each experiment into the base climate and run offline through the Oslo radiative transfer model (Myhre & Stordal, 1997). The difference in TOA fluxes between the two parallel runs constitutes the rapid adjustment. For clouds, we use cloud ice content, cloud water content, cloud fraction on each layer, and total cloud fraction. Cloud droplet effective radius is tuned to obtain net SW downwelling and LW upwelling fluxes close to the observational TOA value of 240 W m^-2^. Five SW calculations per month using Gaussian-quadrature weighted solar zenith angles and one LW calculation are performed. MMPRP calculations are produced without aerosols and ozone present in the radiative transfer code but with model-specific base concentrations of the well-mixed greenhouse gases. It should be noted that monthly mean cloud fields in the MMPRP may provide less accurate estimates of cloud adjustment than using instantaneous climate model output as in a true PRP. This is due to the non-linear absorption and scattering properties of clouds, and monthly averaging may provide large amounts of optically thin cloud which could be an issue particularly for SW radiation (Colman et al., 2001). A true PRP was not performed due to the lack of sub-daily output in most models. Figure S3 shows that the differences between the MMPRP and kernel estimates for water vapor (variable hus) are minimal. There are some differences in some models for cloud adjustment between the MMPRP and kernels, most notably in those models and experiments with a large SW cloud adjustment.

Text S5. Approximate partial radiative perturbation (APRP)

The APRP method can be used to decompose SW cloud responses using only standard CMIP-style diagnostics (Taylor et al., 2007; Zelinka et al., 2014). APRP approximates each model’s radiation code using a single pass through the atmosphere, and tunes the atmospheric absorption, scattering and reflection based on model values of TOA and surface SW fluxes and total cloud fraction. It allows diagnosis of the radiative forcing due to aerosol-radiation interactions (RFari; the direct effect) and aerosol-cloud interactions (RFaci (Twomey, 1977)), and adjustments due to cloud lifetime change (Albrecht, 1989). The APRP method is useful when non-cloud adjustments are small, to avoid aliasing of non-cloud adjustments into RFaci.


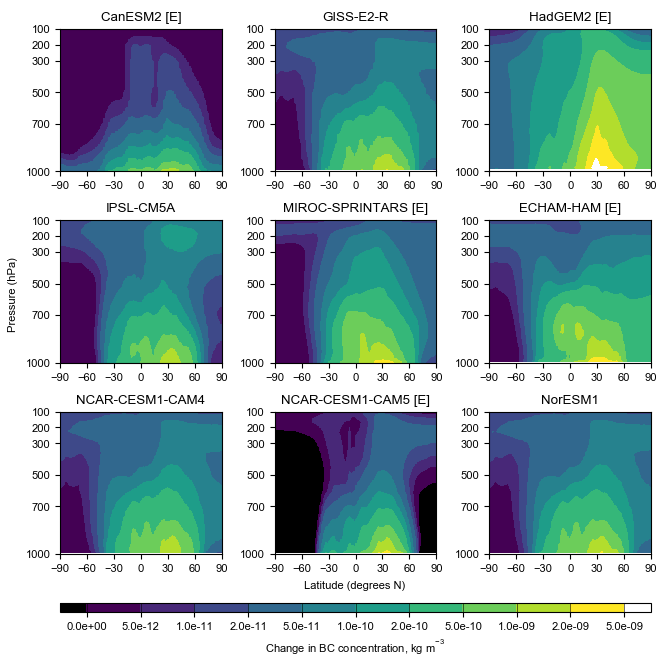


Figure S1. Change in BC mass mixing ratio for the 10xBC experiments (HadGEM3 data not available but vertical profile shown to be almost identical to GISS-E2-R and NorESM1 in Stjern et al. (2017)). Emissions-driven models are marked with [E].


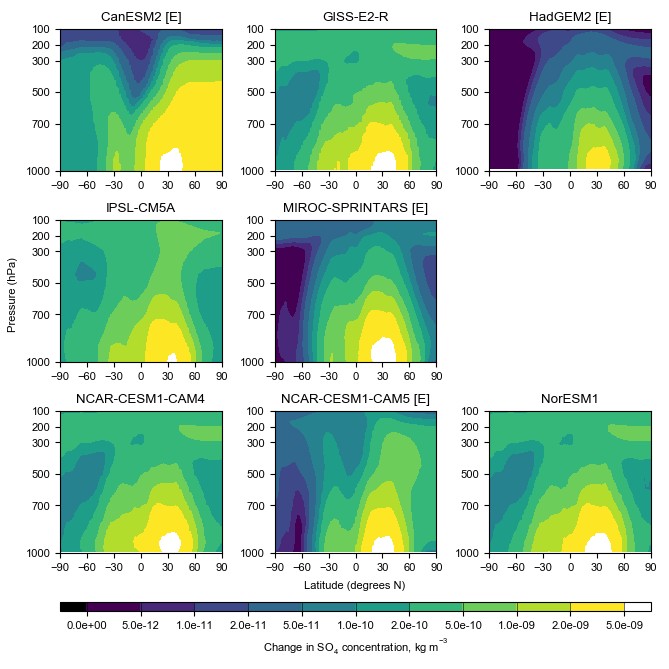


Figure S2. Change in SO_4_ mass mixing ratio for the 5xSul experiments (HadGEM3 data not available but assumed very similar to GISS-E2-R, NCAR-CESM1-CAM4 and NorESM1). Emissions-driven models are marked with [E].


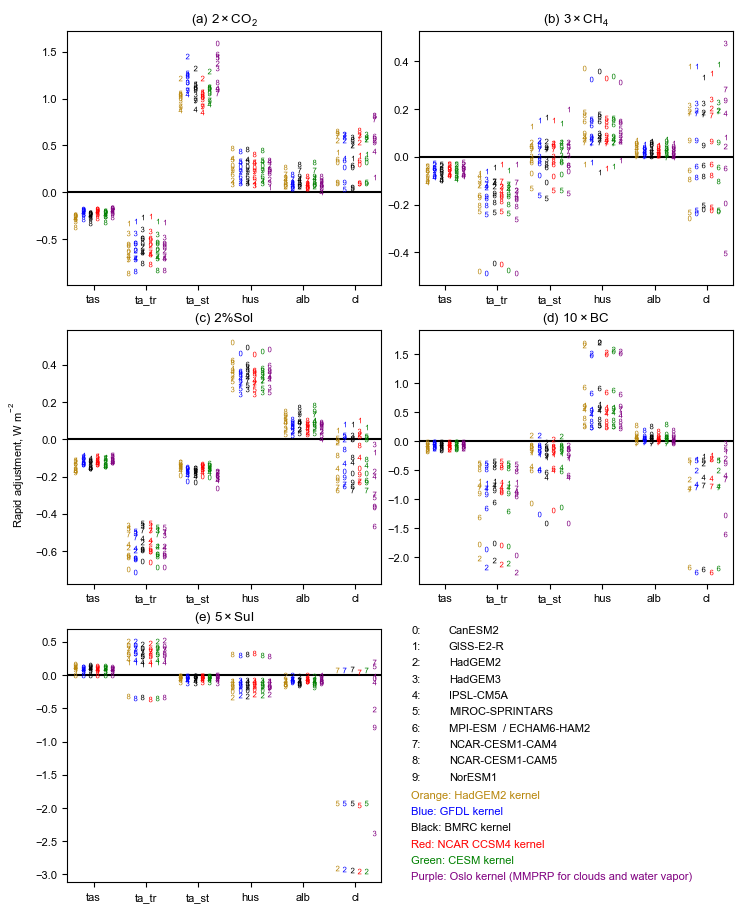


Figure S3. Rapid adjustments for all models and all calculation methods. Plot shows models and methods where sufficient data was available to calculate the adjustments. Adjustments are shown for surface temperature (tas), tropospheric temperature (ta_tr), stratospheric temperature (ta_st), water vapor (hus), surface albedo (alb) and clouds (cl). Cloud adjustments can be calculated from kernels if all-sky and clear-sky IRF is known or can be easily estimated. For 5xSul, the “adjustments” for HadGEM2-ES and MIROC-SPRINTARS both contain a component of RFaci (cloud-albedo effect), and the kernel methods cannot distinguish this from the true adjustment. These results are shown here for comparison, but not included in the multi-model comparisons of cloud adjustments for 5xSul (Figures 1 and 3).


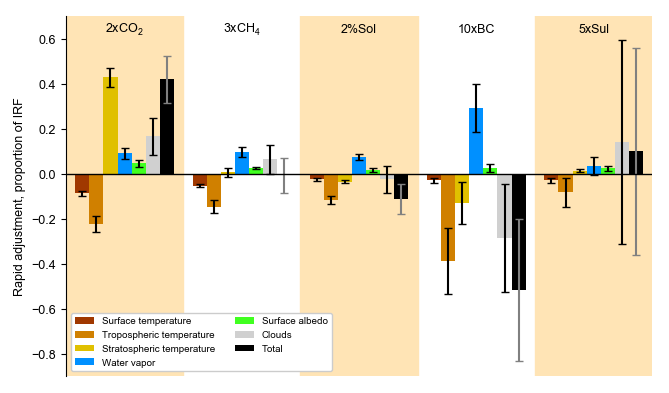


Figure S4. Rapid adjustments for each component normalized by IRF. Compare Figure 3 in main paper.


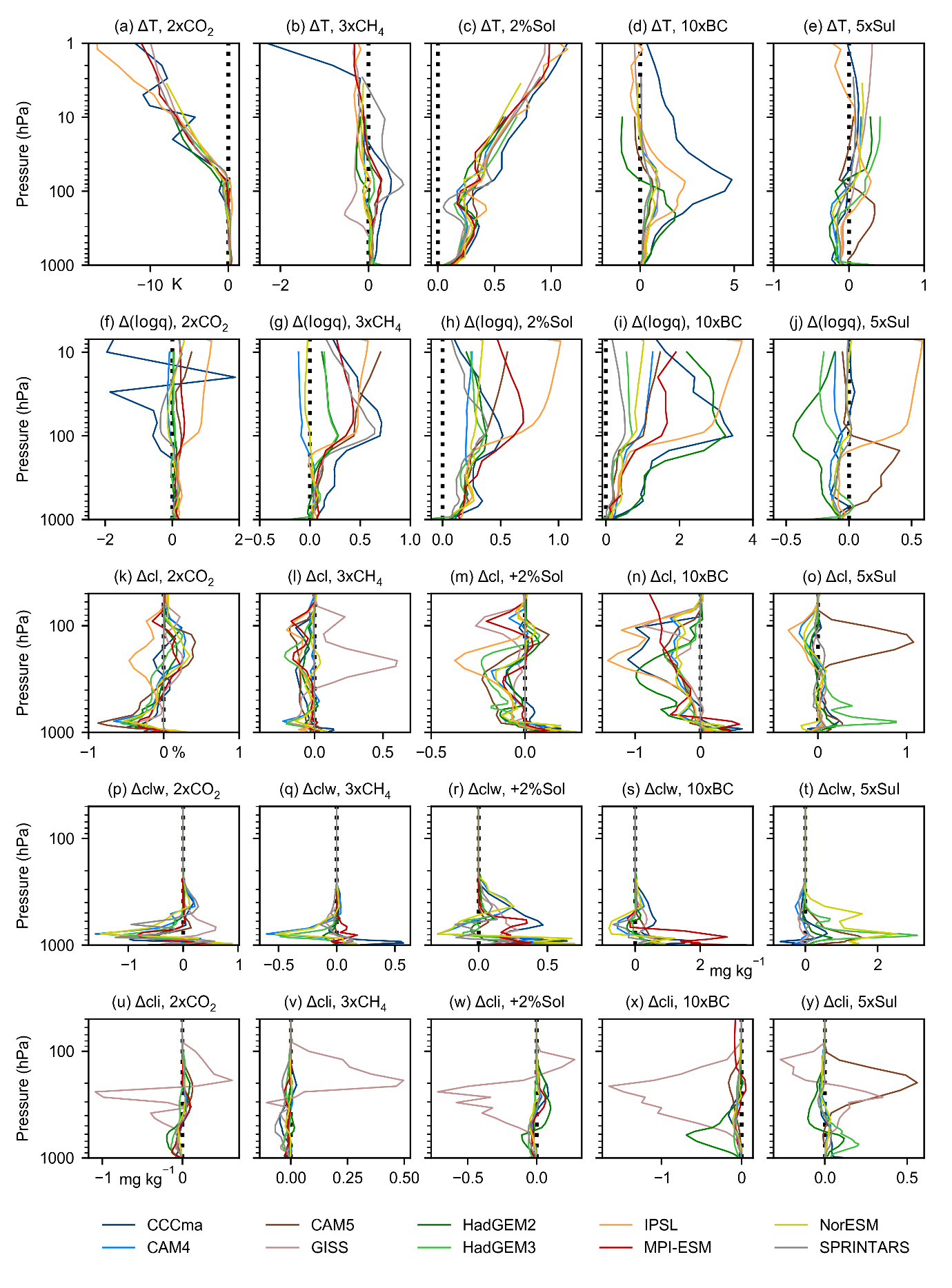


Figure S5. Changes in vertical profile in (a-e) temperature, (f-j) specific humidity, (k-o) cloud fraction, (p-t) cloud water content and (u-y) cloud ice content in each model and experiment. Plots for each model and each experiment show the perturbed profile compared to the control. Note different scales on axes between adjustments and mechanisms.


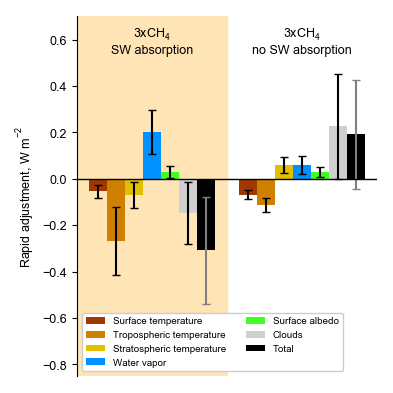


Figure S6. Rapid adjustments by mechanism for models including and excluding an explicit treatment of SW CH_4_ absorption. SW absorption of methane is included in CanESM2, MIROC-SPRINTARS, MPI-ESM and NCAR-CESM1-CAM5.


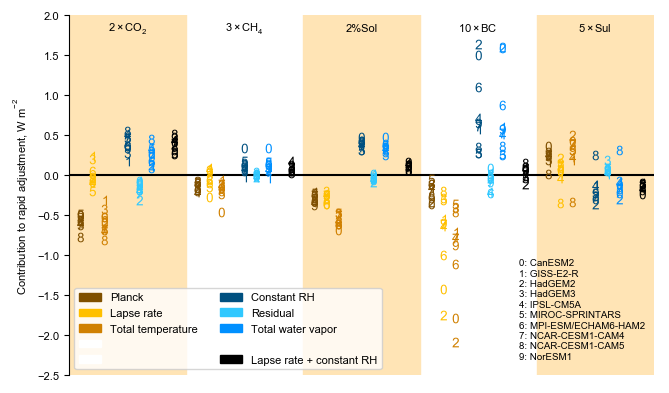


Figure S7. Decomposition of tropospheric temperature and water vapor adjustments for each model (average over kernels). Tropospheric temperature adjustment is decomposed into a Planck component, which assumes a uniform vertical change in tropospheric temperature change, and a lapse rate component, which is a measure of how the change in vertical heating profile affects TOA radiation. Water vapor is decomposed into a constant relative humidity (RH) component and a “residual” which is the difference between the total kernel-calculated water vapor adjustment and the water vapor adjustment calculated assuming constant RH.


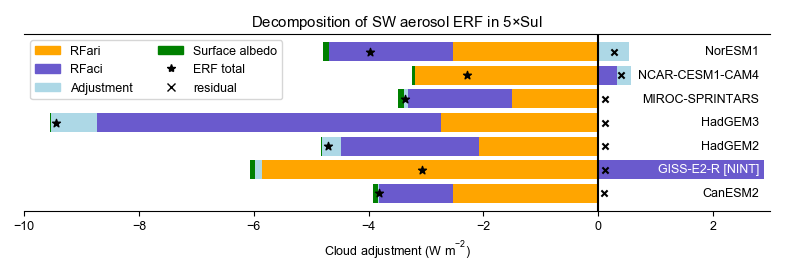


Figure S8. Approximate Partial Radiative Perturbation in 5xSul. Results shown from models with sufficient diagnostics to calculate cloud adjustments. The total ERF (black stars) are decomposed into contributions from RFari (orange; direct forcing), RFaci (purple), rapid adjustments (pale blue) and surface albedo (green). The residual (black cross) is the difference between the ERF and the sum of the constituent components.


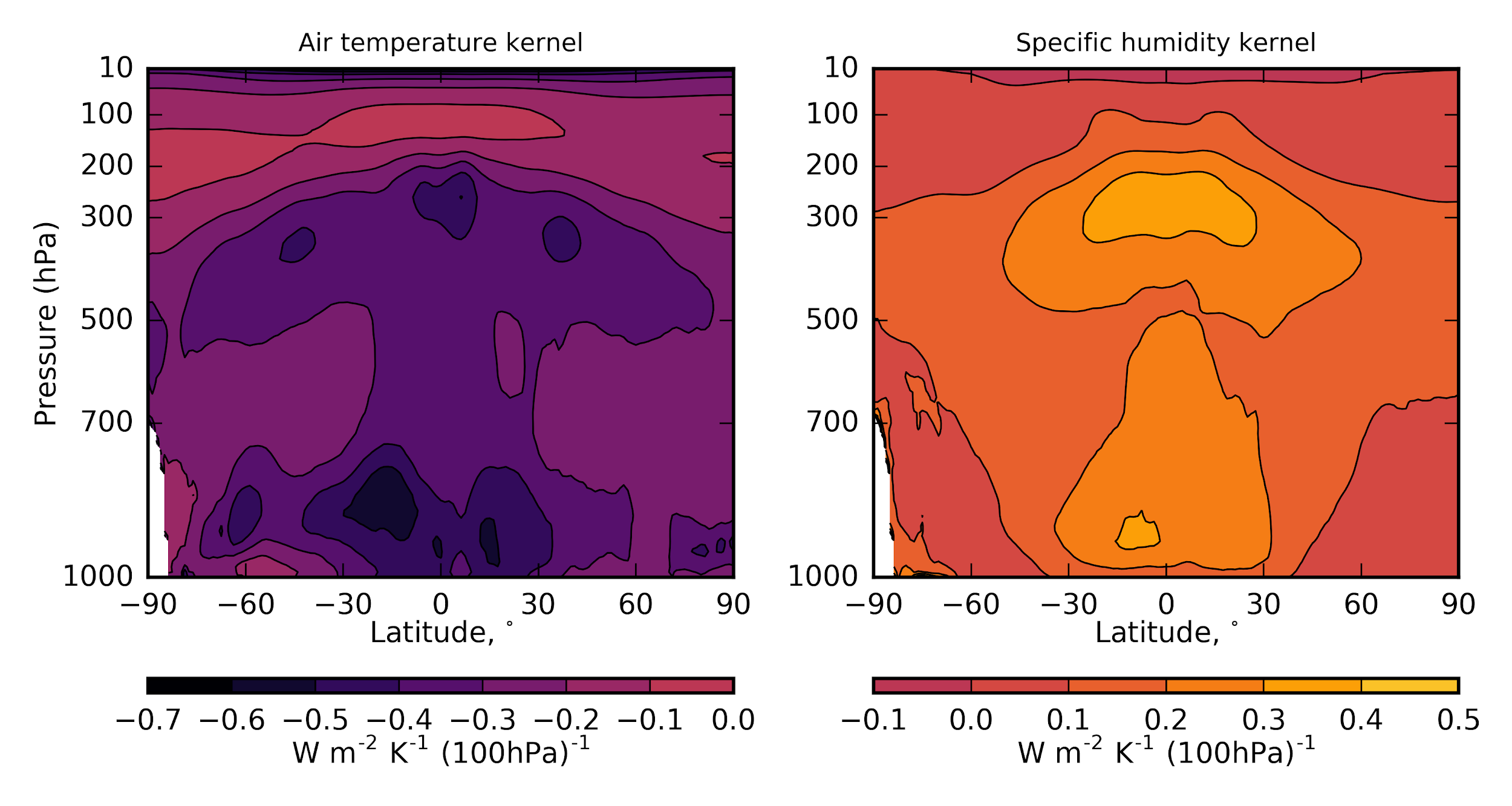


Figure S9. 4-dimensional kernels derived from the HadGEM2 model. Left panel: change in net TOA flux for a 1 K perturbation in atmospheric temperature for each latitude/height (the 4D kernel is shown averaged over time and longitude). Right panel: change in net TOA flux for a unit of specific humidity increase consistent with a 1 K temperature increase.


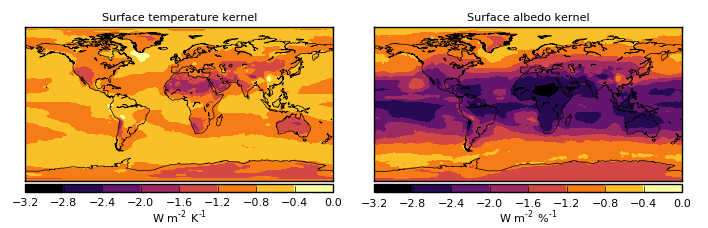


Figure S10. 3-dimensional kernels derived from the HadGEM2 model. Left panel: net change in TOA flux for a 1 K increase in surface temperature. Right panel: net change in TOA flux for a 1% absolute increase in surface albedo.

| Model identifier and name | Climatology | Aerosol treatment | Experiments | Double calls from model’s native radiation code(a) | Explicit treatment of SW CH_4_ absorption in radiation code | Explicit treatment of LW BC absorption in radiation code | Number of different adjustment estimates |
| --- | --- | --- | --- | --- | --- | --- | --- |
| 0. CanESM2 | 1850 | Emissions | 2xCO_2_, 3xCH_4_, 10xBC, 5xSul, 2%Sol |  | Yes | Yes | 6 (1 for 10xBC and 5xSul clouds) |
| 1. GISS-E2-R | 2010 | Concentrations | 2xCO_2_, 3xCH_4_, 10xBC, 5xSul, 2%Sol | 10xBC | No | Yes | 6 (1 for 5xSul clouds) |
| 2. HadGEM2-ES | 1860 | Emissions | 2xCO_2_, 3xCH_4_, 10xBC(b), 5xSul(b), 2%Sol | 2xCO2, 3xCH4, 10xBC, 5xSul | No | Yes | 6 (1 for 5xSul clouds) |
| 3. HadGEM3 | 2000 | Concentrations | 2xCO_2_, 3xCH_4_, 10xBC, 5xSul, 2%Sol |  | No | Yes | 6 (1 for 10xBC and 5xSul clouds) |
| 4. IPSL-CM5A | 2000 | Concentrations | 2xCO_2_, 3xCH_4_, 10xBC, 5xSul, 2%Sol | 10xBC | No(c) | No | 6 (1 for 2xCO_2_, 3xCH_4_, 5xSul clouds) (d) |
| 5. MIROC-SPRINTARS | 2000 | Emissions | 2xCO_2_, 3xCH_4_, 10xBC, 5xSul, 2%Sol | 10xBC, 5xSul | Yes | Yes | 6 (1 for 5xSul clouds) |
| 6. MPI-ESM | 2000 | Concentrations | 2xCO_2_, 3xCH_4_, 2%Sol |  | Yes |  | 6 (2xCO2, 3xCH4 and 2%Sol) |
| 6. ECHAM6-HAM2 (e) | 2000 | Emissions | 10xBC | 10xBC |  | Yes | 6 (10xBC only) |
| 7. NCAR-CESM1-CAM4 | 2000 | Concentrations | 2xCO_2_, 3xCH_4_, 10xBC, 5xSul, 2%Sol | 2xCO_2_, 3xCH_4_, 10xBC, 5xSul | No | No | 6 |
| 8. NCAR-CESM1-CAM5 | 2005 | Emissions | 2xCO_2_, 3xCH_4_, 10xBC, 5xSul, 2%Sol |  | Yes | Yes | 5 (0 for 10xBC and 5xSul clouds) (f) |
| 9. NorESM1 | 2000 | Concentrations | 2xCO_2_, 3xCH_4_, 10xBC, 5xSul, 2%Sol |  | No | No | 6 (1 for 10xBC and 5xSul clouds) |

a. 2%Sol IRF calculated in all models except NCAR-CESM1-CAM5 and independently of radiation code using eq. (2).

b. HadGEM2-ES uses year-1860 base aerosol emissions, except in 10xBC or 5xSul where the perturbation is 10x or 5x year-2000 emissions.

c. A simplified shortwave absorption for a combination of CO_2_ and CH_4_ is present, but does not isolate the effects of CH­_4_ explicitly.

d. clw and cli variables not available, preventing offline SOCRATES estimate of IRF for 2xCO_2_ and 3xCH_4_ and hence kernel-based cloud adjustment methods not available.

e. the ECHAM6-HAM2 model is sufficiently similar to MPI-ESM to be grouped together, as they run different experiments. The models share the same ECHAM base model but ECHAM6-HAM2 has the microphysical aerosol model HAM and different two-moment cloud microphysics.
f. No clt variable available preventing use of the PRP for cloud adjustments or calculation of 2%Sol IRF.

Table S1. Summary of models used in this study. Model numbers refer to Figure 2 and Figures S3 and S7.

# References

Albrecht, B. A. (1989). Aerosols, cloud microphysics, and fractional cloudiness. *Science, 245*(4923), 1227-1231.

Alduchov, O. A., & Eskridge, R. E. (1996). Improved Magnus form approximation of saturation vapor pressure. *Journal of Applied Meteorology, 35*, 601-609.

Block, K., & Mauritsen, T. (2013). Forcing and feedback in the MPI-ESM-LR coupled model under abruptly quadrupled CO_2_. *Journal of Advances in Modeling Earth Systems, 5*, 691-696.

Chung, E.-S., & Soden, B. J. (2015). An assessment of methods for computing radiative forcing in climate models. *Environmental Research Letters, 10*(7), 074004. <http://stacks.iop.org/1748-9326/10/i=7/a=074004>

Collins, W. D., Ramaswamy, V., Schwarzkopf, M. D., Sun, Y., Portmann, R. W., Fu, Q., et al. (2006). Radiative forcing by well‐mixed greenhouse gases: Estimates from climate models in the Intergovernmental Panel on Climate Change (IPCC) Fourth Assessment Report (AR4). *Journal of Geophysical Research: Atmospheres, 111*(D14). <https://agupubs.onlinelibrary.wiley.com/doi/abs/10.1029/2005JD006713>

Colman, R., Fraser, J., & Rotstayn, L. (2001). Climate feedbacks in a general circulation model incorporating prognostic clouds. *Clim. Dynam., 18*(1), 103-122.

Edwards, J. M., & Slingo, A. (1996). Studies with a flexible new radiation code. I: Choosing a configuration for a large-scale model. *Q. J. Roy. Meteor. Soc., 122*(531), 689-719.

Huang, Y., & Bani Shahabadi, M. (2014). Why logarithmic? A note on the dependence of radiative forcing on gas concentration. *Journal of Geophysical Research: Atmospheres, 119*(24), 13,683-613,689. <https://agupubs.onlinelibrary.wiley.com/doi/abs/10.1002/2014JD022466>

Manners, J., Edwards, J. M., Hill, P., & Thelen, J.-C. (2015). SOCRATES (Suite Of Community RAdiative Transfer codes based on Edwards and Slingo) Technical Guide. In: Met Office, UK.

Martin, G. M., Bellouin, N., Collins, W. J., Culverwell, I. D., Halloran, P. R., Hardiman, S. C., et al. (2011). The HadGEM2 family of Met Office Unified Model climate configurations. *Geosci. Model Dev., 4*(3), 723-757. <http://www.geosci-model-dev.net/4/723/2011/>

Pendergrass, A. G., Conley, A., & Vitt, F. M. (2018). Surface and top-of-atmosphere radiative feedback kernels for CESM-CAM5. *Earth Syst. Sci. Data, 10*(1), 317-324. <https://www.earth-syst-sci-data.net/10/317/2018/>

Sanderson, B. M., & Shell, K. M. (2012). Model-Specific Radiative Kernels for Calculating Cloud and Noncloud Climate Feedbacks. *Journal of Climate, 25*(21), 7607-7624. <https://journals.ametsoc.org/doi/abs/10.1175/JCLI-D-11-00726.1>

Shell, K. M., Kiehl, J. T., & Shields, C. A. (2008). Using the Radiative Kernel Technique to Calculate Climate Feedbacks in NCAR's Community Atmospheric Model. *J. Climate, 21*, 2269-2282.

Soden, B. J., Held, I. M., Colman, R., Shell, K. M., Kiehl, J. T., & Shields, C. A. (2008). Quantifying Climate Feedbacks Using Radiative Kernels. *J. Climate, 21*(14), 3504-3520.

Stjern, C. W., Samset, B. H., Myhre, G., Forster, P. M., Hodnebrog, Ø., Andrews, T., et al. (2017). Rapid Adjustments Cause Weak Surface Temperature Response to Increased Black Carbon Concentrations. *Journal of Geophysical Research: Atmospheres, 122*(21), 11,462-411,481. <https://agupubs.onlinelibrary.wiley.com/doi/abs/10.1002/2017JD027326>

Taylor, K. E., Crucifix, M., Braconnot, P., Hewitt, C. D., Doutriaux, C., Broccoli, A. J., et al. (2007). Estimating Shortwave Radiative Forcing and Response in Climate Models. *Journal of Climate, 20*(11), 2530-2543. <https://journals.ametsoc.org/doi/abs/10.1175/JCLI4143.1>

Taylor, K. E., Stouffer, R. J., & Meehl, G. A. (2012). An Overview of CMIP5 and the Experiment Design. *B. Am. Meteorol. Soc., 93*(4), 485-498.

Twomey, S. (1977). The influence of pollution on the shortwave albedo of clouds. *Journal of the Atmospheric Sciences, 34*(7), 1149-1152.

Wetherald, R. T., & Manabe, S. (1988). Cloud Feedback Processes in a General Circulation Model. *Journal of the Atmospheric Sciences, 45*(8), 1397-1416. <http://dx.doi.org/10.1175/1520-0469(1988)045%3C1397:CFPIAG%3E2.0.CO;2>

Zelinka, M. D., Andrews, T., Forster, P. M., & Taylor, K. E. (2014). Quantifying components of aerosol‐cloud‐radiation interactions in climate models. *Journal of Geophysical Research: Atmospheres, 119*(12), 7599-7615. <https://agupubs.onlinelibrary.wiley.com/doi/abs/10.1002/2014JD021710>
